# Human Cytomegaloviruses Expressing Yellow Fluorescent Fusion Proteins - Characterization and Use in Antiviral Screening

**DOI:** 10.1371/journal.pone.0009174

**Published:** 2010-02-11

**Authors:** Sarah Straschewski, Martin Warmer, Giada Frascaroli, Heinrich Hohenberg, Thomas Mertens, Michael Winkler

**Affiliations:** 1 Institute of Virology, Ulm University Hospital, Ulm, Germany; 2 Institute for Infection Medicine, Universitätsklinikum Schleswig-Holstein Campus Kiel, Kiel, Germany; 3 Heinrich-Pette-Institute for Experimental Virology and Immunology, University of Hamburg, Hamburg, Germany; Institut Pasteur Korea, Republic of Korea; Institut Pasteur Korea, Republic of Korea

## Abstract

Recombinant viruses labelled with fluorescent proteins are useful tools in molecular virology with multiple applications (e.g., studies on intracellular trafficking, protein localization, or gene activity). We generated by homologous recombination three recombinant cytomegaloviruses carrying the enhanced yellow fluorescent protein (EYFP) fused with the viral proteins IE-2, ppUL32 (pp150), and ppUL83 (pp65). In growth kinetics, the three viruses behaved all like wild type, even at low multiplicity of infection (MOI). The expression of all three fusion proteins was detected, and their respective localizations were the same as for the unmodified proteins in wild-type virus–infected cells. We established the in vivo measurement of fluorescence intensity and used the recombinant viruses to measure inhibition of viral replication by neutralizing antibodies or antiviral substances. The use of these viruses in a pilot screen based on fluorescence intensity and high-content analysis identified cellular kinase inhibitors that block viral replication. In summary, these viruses with individually EYFP-tagged proteins will be useful to study antiviral substances and the dynamics of viral infection in cell culture.

## Introduction

The human cytomegalovirus (HCMV) is a herpesvirus belonging to the *Betaherpesvirinae* subfamily. With a genome size of approximately 235 kbp it contains one of the largest genomes among viruses. HCMV is known to have a narrow host range infecting only humans, where 40–90% of the global human population become seropositive after primary infection. Postnatal primary infection is usually asymptomatic in immune competent hosts and is followed by a lifelong persistence. In individuals with an immature or compromised immune system HCMV can cause considerable morbidity and mortality [1], [2].

The replication cycle of HCMV in the host can be divided into three different phases, the IE-phase, where mainly genes with regulatory functions are expressed, the early-phase for the expression of enzymatic proteins and the late-phase for expression of the structural components [3], [4]. A prominent gene product of the immediate-early phase is the IE2 protein, which is essential for viral replication and functions as activating and repressing transcription factor [5]–[8]. The expression of the structural proteins ppUL83 (pp65) and ppUL32 (pp150) starts during the early and late phase, respectively. The lower matrix protein ppUL83 is a non-essential protein for replication in fibroblasts but important for replication in macrophages [9], [10]. It first accumulates in the nucleus of infected cells but is translocated to the cytoplasm in the late phase in a process which depends on cyclin-dependent kinases and the Crm1 exporter [11]. As a component of the tegument, ppUL83 is located inside the virion but is also the main component of dense bodies [12]. About 20% of the protein components of viral particles comprise ppUL83 which is the main acceptor for phosphate during viral replication [4]. The true late protein ppUL32 (pp150) is an essential protein [4], [13], [14]. This basic phosphoprotein is also a major component of the tegument and is able to bind to the viral capsid [12], [15].

Recombinant fluorescent viruses have been generated for other viruses to investigate the movement of viral particles inside the infected cell [16]–[21]. For HCMV several studies have been published showing ectopic expression of the green fluorescent protein [22]–[24]. In addition, fusions of EGFP with the IE2 protein [25] and the structural protein ppUL32 [26] have been reported. In the present study we used a variant of *Aequorea victoria* green fluorescent protein (GFP) for the labelling of viral proteins. The enhanced yellow fluorescent protein (EYFP) is a mutant of the *av*EGFP with an emission maximum of 530 nm instead of 509 nm for the EGFP [27]. Compared to EGFP the EYFP has a higher absorption and quantum yield [28]. This shift in emission causes an improved intensity of the EYFP compared to EGFP, and the relative auto-fluorescence of the cells is strongly reduced. The reduction of the auto-fluorescence is very important for the use of these fluorescent viruses. In addition, fusions with EYFP have been used in conjunction with ECFP-fusions for FRET measurements to monitor protein-protein interactions in viable intact cells [29].

In this study we generated by recombineering (genetic engineering by recombination [30], [31]) modified HCMV viruses with EYFP fused to the immediate-early protein IE2, the early-late protein ppUL83 and the true late protein ppUL32. First, we characterized expression and localization of the modified proteins in comparison with the wild type proteins. Having done this, we evaluated our fluorescent viruses in a relevant application, namely the screening of antiviral compounds in intact cells. We show here, that the recombinant viruses can be used to quantify the inhibition of viral replication by neutralizing antibodies or to screen for antiviral substances by measuring the fluorescence intensity in intact cells.

## Results

### Generation of EYFP-Expressing HCMV

**Figure 1:**
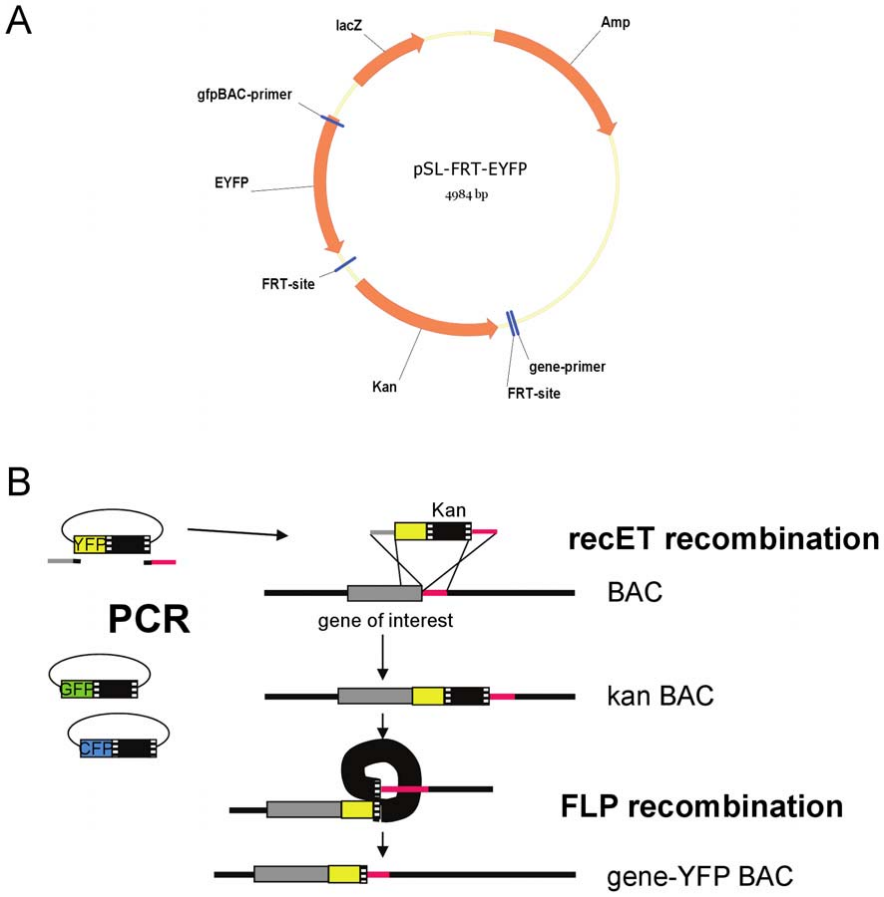
Construction of recombinant HCMV TB40E-EYFP. (**A**) Map of the pSL-FRT-EYFP plasmid. Open reading frames and FRT-sites are indicated. Primer binding sites used for amplification were gfpBAC-primer and gene-primer. (**B**) Scheme of ET-cloning. The EYFP/Kan-cassette was amplified by PCR and used for recET-based recombination to fuse EYFP to the C-terminus of the gene of interest. This resulted in a BAC still containing the kanamycin resistance gene (kan BAC). By transformation of a plasmid encoding the FLP recombinase the kanamycin resistance was removed, leaving a BAC with gene-EYFP fusion and a FRT-site (gene-YFP BAC).

To generate virus variants encoding fusions of viral proteins with EYFP we first generated a plasmid template suitable for recombineering. Plasmid pSL-FRT-EYFP contained the EYFP gene adjacent to a kanamycin resistance gene, which was flanked by FRT sites to allow subsequent removal (Fig. 1A). We chose to generate fusions with an immediate-early (IE2), a delayed-early (ppUL83) and a true late (ppUL32) gene in the context of strain TB40E-BAC4 (thereafter called TB4). The EYFP-kanamycin region was amplified with primers containing sequences homologous to the respective insertion sites (Fig. 1B). Primers were designed to remove the stop codon of the viral gene. In addition, a GPGP-linker peptide was encoded by the fusion primer between the last codon of the viral gene and the first EYFP codon. This peptide should ensure conformational uncoupling between the viral protein and EYFP. After successful recombination the kanamycin resistance cassette was removed by FLP-based recombination leaving the fused EYFP gene and one FRT-site behind.

**Figure 2:**
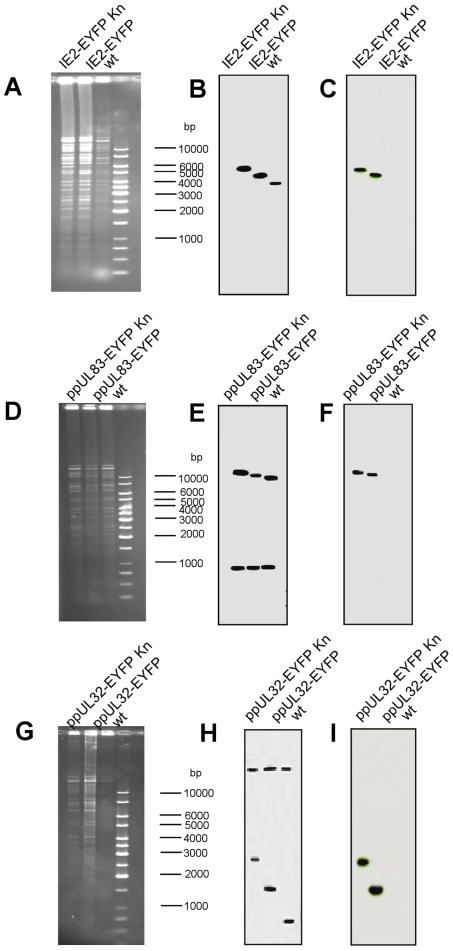
Demonstration of the genomic rearrangement by Southern blot analysis. Respective recombinant BAC DNAs were digested with EcoRV and separated by agarose gel electrophoresis (A), (D) and (G). A size marker is shown on the gel with the fragment sizes indicated on the right side. Subsequently the gel was blotted and subjected to southern hybridization analysis using the [^32^P]dCTP labelled gene-specific (B, E and H) detailed below or an EYFP-specific probe (C, F and I). (**A**) BAC DNA of HCMV TB4-IE2-EYFP-kana^+^ (lane 1), TB4-IE2-EYFP-kana^−^ (lane 2) and TB4wt (lane 3). (**B**) Southern analysis using UL123 (IE2)- and UL120-specific probes. (**D**) BAC DNA of HCMV TB4-UL83-EYFP-kana^+^ (lane 1), TB4-UL83-EYFP-kana^−^ (lane 2) and TB4wt (lane 3). (**E**) Southern blot using UL83- and UL82-specific probes. (**G**) BAC DNA of HCMV TB4-UL32-EYFP-kana^+^ (lane 1), TB4 UL32-EYFP-kana^−^ (lane 2) and TB4wt (lane 3). (**H**) Southern analysis using UL32- and UL31-specific probes.

The recombinant BAC genomes were analyzed by restriction digest and southern blot analysis, using probes of the regions flanking the insertion sites. As shown in Fig. 2A, D, G no unexpected alterations of the genomes were observed. Specific alterations of certain restriction fragments corresponded to fragments altered by insertion of EYFP, as shown in Fig. 2. Specifically, insertion of the EYFP-kanamycin cassette increased the fragments by 2 kb and this was subsequently reduced by 1.1 kb after removal of kanamycin. In the case of TB4-IE2-EYFP, the flanking probes (Fig. 2B) detected a single fragment of 4 (lane 3), 6 (lane 1) and 5 kb (lane 2), respectively, corresponding to wild type or recombinant viruses with or without kanamycin, respectively. Accordingly, the 6 and 5 kb fragments were also detected with the EYFP-specific probe, as expected (Fig. 2C). In the case of TB4-UL83-EYFP, the flanking probes (Fig. 2E) detected two bands of 1 and 10 kb in the wild type virus, were the 10 kb fragment was increased to 12 or 11 kb in the recombinant genomes with or without kanamycin, respectively. Again, the EYFP-specific probe (Fig. 2F) detected only the 12 and 11 kb fragments in the recombinant genomes. Finally, the flanking probes for UL32 (Fig. 2H) detected two signals of 0.8 and >20 kb. Here the size of the small fragment increased to 2.5 or 1.4 kb in the recombinant viruses with or without kanamycin; these fragments also contained EYFP (Fig. 2I). In summary, we successfully generated fusions of EYFP to IE2, UL83 and UL32, respectively, in the context of the genome of HCMV strain TB40E.

### No Growth Defect of Variant Viruses

**Figure 3:**
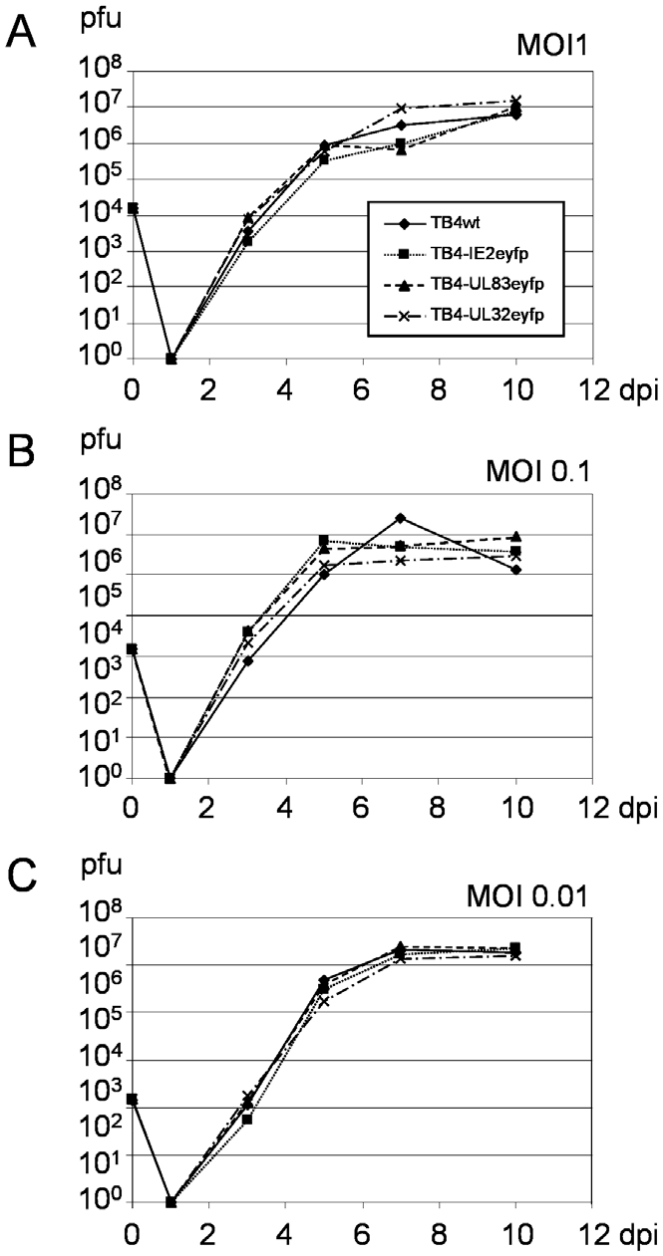
Virus growth kinetics of recombinant viruses in human foreskin fibroblasts. HFF were infected with TB4-wt (circles), TB4-IE2-EYFP (squares), TB4-UL83-EYFP (triangles) and TB4-UL32-EYFP (crosses) at MOI of 1 (**A**), 0.1 (**B**) and 0.01 (**C**). Supernatant was collected and titrated over a range of 10 days post infection.

The recombinant BACs were reconstituted in HFF. To analyze if the fusion of EYFP to the selected viral proteins affected growth in cell culture, we infected human fibroblasts with wild type or modified viruses at an MOI of 1 and the virus yield was determined over a range of 10 days post infection. As shown in Fig. 3A, no significant difference in growth properties was observed between wild type and any of the fluorescent viruses. Since minor growth defects become more apparent upon infection at low MOI [32]–[34], we determined additional one-step growth curves at MOI 0.1 (Fig. 3B) and 0.01 (Fig. 3C). The modified viruses grew like wild type TB4-wt even at the low MOI of 0.01. Since no significant growth defect was observed, these viruses should be useful tools for either analyzing the normal viral life cycle or the inhibition of viral replication by fluorescence microscopy or fluorescence measurement.

### The Fusion Products of Viral Proteins with EYFP Can Be Detected by Western Blot Analysis

**Figure 4:**
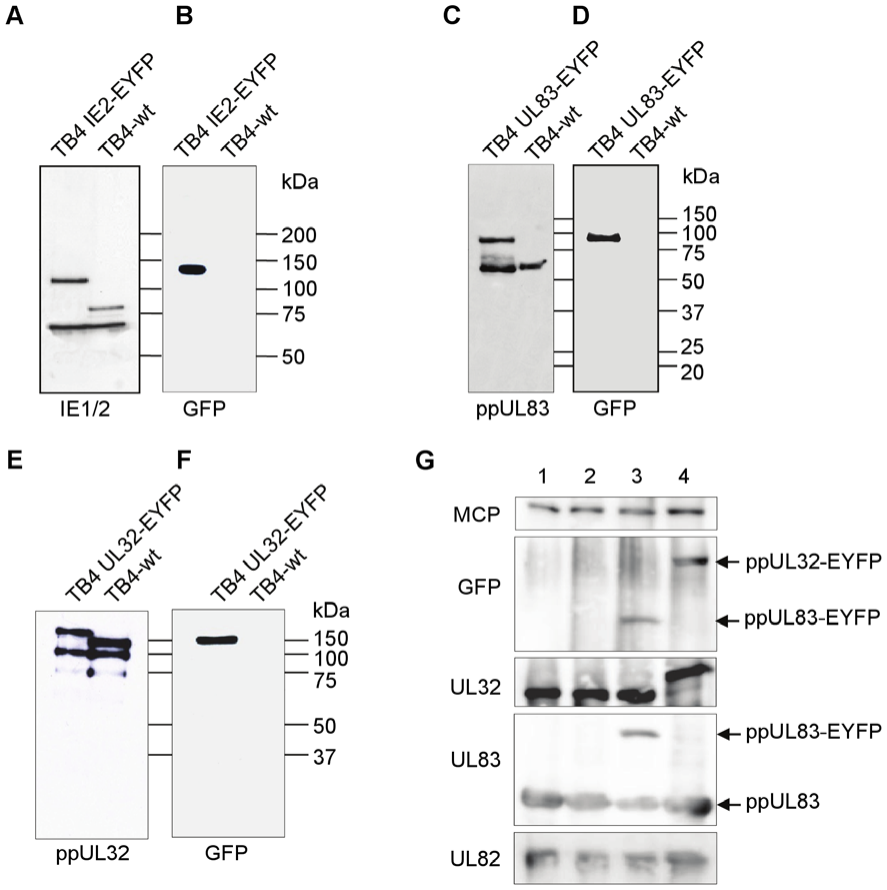
Western blot analysis of recombinant viruses. At 72 hpi, TB4- and recombinant virus-infected HFF (MOI of 1) were harvested, lysed and the proteins were separated by a 10% SDS-PAGE. Detection was done by using specific antibodies against the viral fusion partner or the fluorescent protein. (**A**) detection of IE1+2 with polyclonal rabbit anti-IE1+2. (**B**) anti-GFP antibody. (**C**) detection of ppUL83 and ppUL83-EYFP with specific anti-pp65 or (**D**) anti-GFP antibody. ppUL32 was detected with an specific anti-ppUL32 antibody (**E**) or also with the anti-GFP antibody (**F**). In (**G**) the lysates of cell free viral stocks of TB4-wt (lane 1), TB4-IE2-EYFP (lane 2), TB4-UL83-EYFP (lane 3) and TB4-UL32-EYFP (lane 4) were separated, blotted and detected with antibodies specific for major capsid protein (MCP, pUL86), GFP, ppUL32, ppUL82 and ppUL83. The stocks had been adjusted to equal levels of MCP.

Next, we were interested whether the fusion proteins were correctly synthesized and localized. First, we performed immunoblot analyses of cells infected with the different recombinant viruses. HFF cells were infected with an MOI of 1 and harvested at 24 hpi (IE2-EYFP) or 72 hpi (UL83-EYFP and UL32-EYFP). Fusion proteins were detected using specific antibodies directed against the respective viral protein or GFP. The IE2-EYFP fusion protein could be readily detected as a band of about 115 kDa, both by an IE1/2 specific serum (Fig. 4A) and a GFP-specific serum (Fig. 4B). An additional band at 72 kDa represented the IE1 protein, which was not modified in the TB4-IE2-EYFP virus. In cells infected with TB4-UL83-EYFP two bands of 65 and 95 kDa were detected by a monoclonal antibody directed against ppUL83 (pp65) (Fig. 4C). The 95 kDa band corresponded to the ppUL83-EYFP fusion protein, as demonstrated by staining with the anti-GFP serum (Fig. 4D), whereas the 65 kDa band represented either a product of incomplete synthesis or post-translational processing. However, no free EYFP (expected size 28 kDa) was detected (Fig. 4D), indicating incomplete synthesis. A band of the expected size of 180 kDa was also observed in cells infected with TB4-UL32-EYFP, both with anti-ppUL32 monoclonal antibody (Fig. 4E) and anti-GFP serum (Fig. 4F). Two bands of 100 and 75 kDa represent unspecific signals of the anti-ppUL32 monoclonal antibody.

Since ppUL83 (pp65) and ppUL32 (pp150) represent major components of the virus particle we analyzed concentrated viral particles for the presence of EYFP-fusion proteins in the respective virus variants. Viral particles were standardized for equal amount of major capsid protein (MCP), as shown in Fig. 4G. By immunoblot using an anti-GFP serum both ppUL83-EYFP and ppUL32-EYFP could be readily detected (Fig. 4G). No GFP signals were observed for particles of the wild type virus and the TB4-IE2-EYFP variant. Since IE2 is not incorporated into viral particles but only expressed in infected cells, this also controls for the purity of our particle preparation. When virus particles were probed with antibodies for ppUL83 or ppUL32 similar amounts of proteins were detected in all preparations. Only for TB4-UL83-EYFP there appeared to be less ppUL83-EYFP, which was supplemented by a band at 65 kDa, possibly a product of incomplete synthesis of the fusion product as outlined above. Finally, an additional major tegument protein, ppUL82 (pp71), was also present in all preparations at equal amounts. Thus, all virus variants expressed fusion proteins of the expected size, and the ppUL83- and ppUL32-fusion proteins were correctly incorporated into virus particles.

### Kinetics of Intracellular Localization of Recombinant Viruses Compared to the Wild-Type Virus

**Figure 5:**
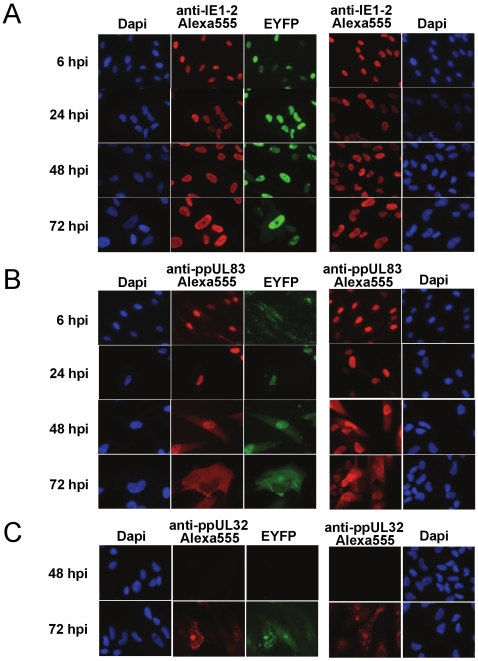
Immunofluorescence analysis of HFF cells infected with recombinant viruses. The co-localisation of viral protein and EYFP in HFF infected with the recombinant virus was detected by fluorescence microscopy at 40× magnification. HFF were infected with TB4-IE2-EYFP (**A**), TB4-UL83-EYFP (**B**) or TB4-UL32-EYFP (**C**) with MOI 1 and fixated at 6, 24, 48 and 72 hpi as indicated on the left side. Cells were stained with mouse monoclonal antibodies directed against IE1–2 (E13) (A), ppUL83 (B) or ppUL32 (XP-1) (C) and Alexa-Fluor555 conjugated secondary antibody. Single channel recordings of DNA (Dapi), EYFP and the viral protein portion (red staining with Alexa-Fluor555) are shown in the left three columns; merged images are in the rightmost column. The time after infection is indicated on the left side.

We then analyzed, if the intracellular localization of the viral EYFP-fusion proteins corresponded to the situation in wild type infected cells. For this, we infected HFF cells with wild type virus and the three different recombinant viruses and performed immunofluorescence analyses at different time points post infection. For TB4-IE2-EYFP a nuclear localization was observed at all time points (Fig. 5A). As in wild type-infected cells, IE2 showed a diffuse nuclear staining at 24 hpi, which developed into a specific subnuclear staining of inclusion bodies later during infection. The staining of an IE2-specific rabbit serum showed clear overlap with EYFP fluorescence. In cells infected with TB4-UL83-EYFP a punctate surface staining from adhered particles was observed 6 hpi along with nuclear accumulation of ppUL83 from infecting particles (Fig. 5B, top row). For ppUL83-EYFP only weak nuclear staining could be detected, potentially due to the fact that only a fraction of ppUL83 in virus particles was fused to EYFP (Fig. 4G). During the early phase a nuclear localization was detected, whereas in the late phase the protein migrated to the cytoplasm (Fig. 5B) as in the wild type virus infected cells. Again a close overlap of EYFP fluorescence and staining by a monoclonal antibody specific for ppUL83 was observed. Finally, ppUL32-EYFP was first detected during the late phase (72 hpi) and was localized towards the periphery of the nucleus and in the cytoplasm throughout the late phase (Fig. 5C). Signals from EYFP fluorescence and staining by a ppUL32 specific monoclonal antibody showed a perfect overlap. The sequence of expression of the fusion-proteins corresponded to their respective gene expression kinetic class, with IE2-EYFP being detected from 6 hpi, ppUL83-EYFP from 24 hpi and ppUL32-EYFP in the late phase at 72 hpi. For all recombinant viruses the EYFP fluorescence signal corresponded to the signal of the respective viral fusion partner and its localization in wild type infected cells.

### Use of Recombinant Viruses for Testing Neutralizing Antibodies

**Figure 6:**
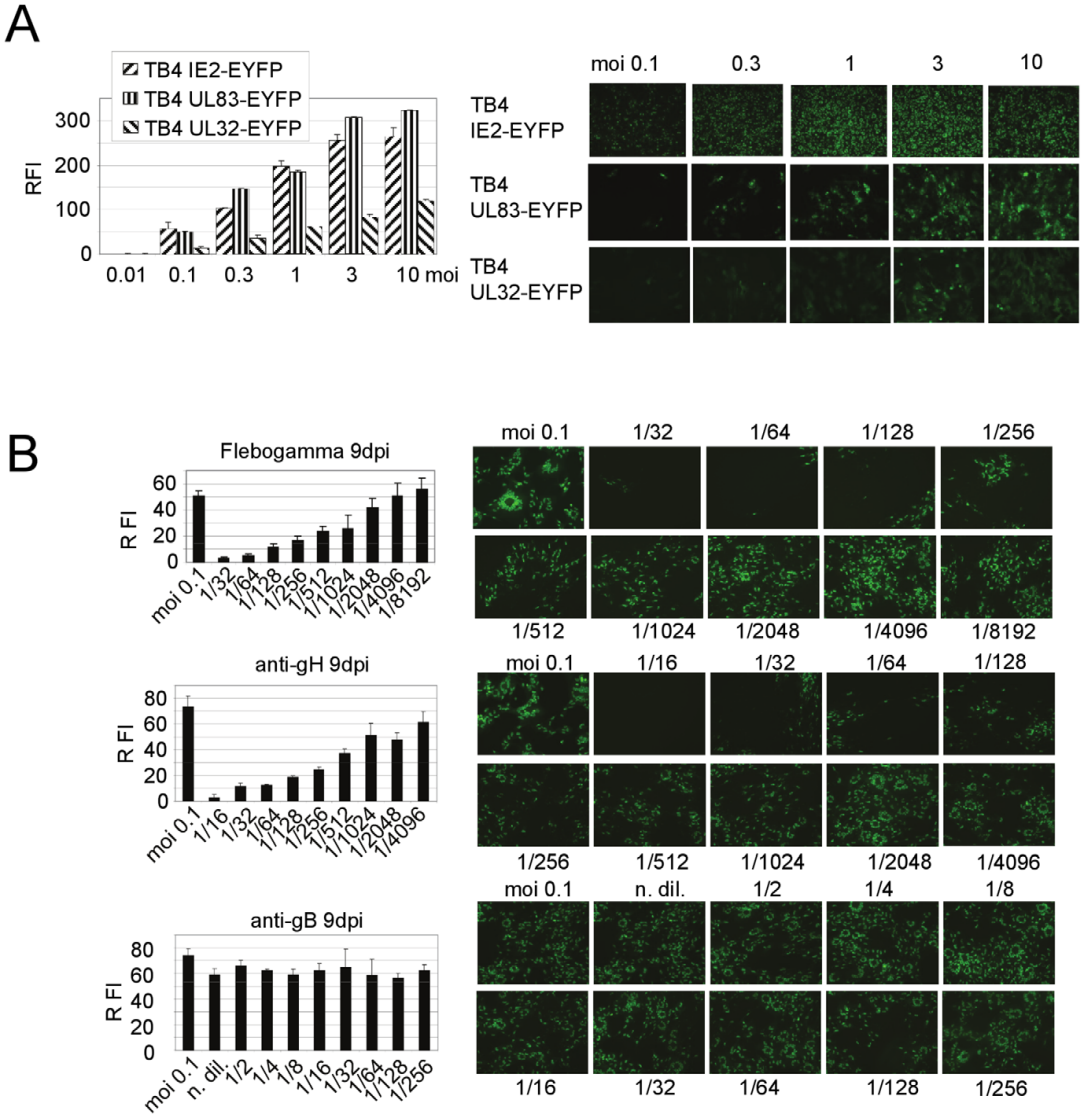
Testing measurement of antiviral agents. HFF infected in 96-well plates and fluorescence was measured in live cells by fluorescence spectrometry or detected by live microscopy at 10-fold magnification. (**A**) The relationship of relative fluorescence intensity (RFI) and multiplicity of infection is shown in the left panel for cells infected with the indicated viruses for 8 days. The panels on the right show cells infected with TB4-IE2-EYFP (5 dpi) and TB4-UL83-EYFP or TB4-UL32-EYFP (8 dpi) at the indicated MOI. (**B**) Measurement of neutralizing activity using TB4-IE2-EYFP. Virus was incubated with different dilutions of Flebogamma^R^ (5%) (upper row) or supernatant from hybridoma cell lines producing antibodies directed against anti-gH (14-4B) (middle row) or anti-gB (27–39) (bottom row). HFF were infected at MOI 0.1 and relative fluorescence intensities recorded at 9 dpi are shown in the panels on the left. Corresponding microscopic images are shown in the panels on the right.

To determine, whether the infection with these viruses could be quantified, we established the infection in the 96-well format and measured the fluorescence signal in live cells using a Cary Eclipse fluorescence spectrophotometer. First, we determined the best time-point for measurement and analyzed the dependence of the fluorescence signal on the MOI used for infection. We infected HFF cells with different MOI ranging from 0.01 to 10 and measured fluorescence intensity in arbitrary units. We obtained the best results 8 dpi, which was chosen for further measurements. As shown in Fig. 6A all viruses showed a nearly linear correlation between fluorescence signal and the viral MOI over about two orders of magnitude. The fluorescence signal intensities for TB4-IE2-EYFP and TB4-UL83-EYFP were similar and 3-4-fold higher than for TB4-UL32-EYFP.

To evaluate the use of these viruses for measuring virus inhibitors, we first used the TB4-IE2-EYFP to measure inhibition of virus entry by neutralizing antibodies. For these experiments we chose to infect cells with MOI of 0.1. Virus was preincubated with Flebogamma^R^, a commercial hyperimmune globulin preparation, a neutralizing antibody directed against glycoprotein H (14-4B) and a non-neutralizing antibody (27–287) directed against glycoprotein B. As demonstrated in Fig. 6B, the inhibition by Flebogamma^R^ and the neutralizing antibody was readily detected. The EC50 for Flebogamma^R^ was determined at a dilution of 1/1024 and for anti-gH the EC50 was at a dilution of 1/512. These results were also confirmed by fluorescence microscopy. As control, after preincubation with different concentrations of the non neutralizing anti-gB antibody no reduction in fluorescence intensity was observed.

In additional experiments we evaluated the effect of GCV on the replication of TB4-IE2-EYFP, TB4-UL83-EYFP and TB4-UL32-EYFP. These measurements were performed on a Tecan Safire 2, which allowed us to detect signals as early as 4 dpi. Using the three viruses we established EC50 values for GCV of 5.43±0.15 µM, 7.58±0.39 µM and 8.09±0.06 µM, respectively. These experiments demonstrate the use of these viruses to quantify infection and antiviral activities.

### Use of Recombinant Viruses for Antiviral Screening

**Figure 7:**
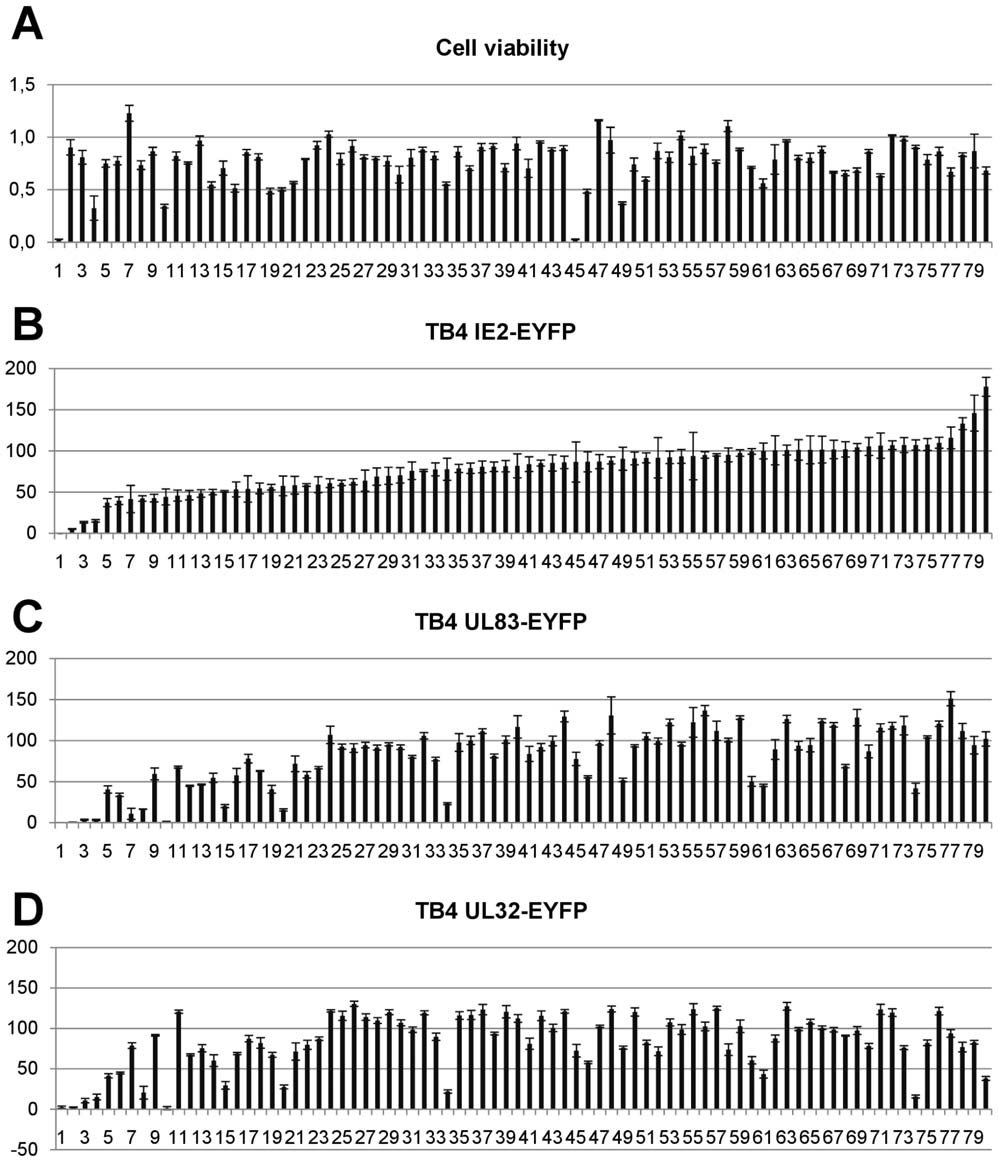
Screening a kinase inhibitor library. A kinase inhibitor library (lanes 1–80) was used to measure cell viability (A) or inhibition of virus replication using TB4-IE2-EYFP (B), TB4-UL83-EYFP (C) or TB4-UL32-EYFP (D). All measurements were done at 4 dpi. (A) To determine cell viability all cells remained uninfected. Cell viability of mock infected cells was set 1.0. (B)–(D) Fluorescence intensity was measured in a Tecan Safire 2. Background (uninfected cells) was subtracted from all samples and normalized to infected cells (100%). Inhibitors were ordered according to the inhibition of TB4-IE2-EYFP.

To demonstrate the feasibility of antiviral screening with these viruses, we used a library of cellular kinase inhibitors. As before infections were performed in the microplate format and measured in the Tecan Safire 2 microplate reader at 4 dpi. Inhibitors were added at a concentration of 10 µM at the time of infection and replaced every second day. We first evaluated the toxicity of the compounds in HFF used in our experiments (Fig. 7A). In several samples cells died (samples 1, 45) or were severely compromised (less than 50% viability; samples 4, 10, 49) indicating that the inhibitors were added at a toxic concentration. For cells treated with most inhibitors or different concentrations GCV the viability was above 50%. Among the kinase inhibitors showing a cell viability of 80–90% at the drug concentration used, we observed with all viruses a strong inhibition (less than 25% fluorescence intensity) for two compounds (Rottlerin and Tyrphostin 9, samples 2 and 3) (Fig. 7B). An additional compound (Ro 31–8220, sample 4) showed strong inhibition but also a severely compromised viability (33%), while Staurosporine (sample 1) was toxic for all cells. Thus, we could clearly show that the fluorescent viruses can be used to identify inhibitory compounds in a high throughput screening system.

When we compared whether the three fluorescent viruses produced similar results we observed, that some compounds produced differential pattern of inhibition. Two compounds (KN-93 and Kenpaullone, samples 15 and 74) showed a more pronounced inhibition of TB4-UL83-EYFP or TB4-UL32-EYFP compared to TB4-IE2-EYFP with a high viability of the non-infected cells, while others (samples 5-Iodotubercidine, GF-109203X or BML-265, samples 10, 20 and 34) had a more reduced cell viability (30–50%). These compounds could be involved in the regulation of effects selective for specific phases of the replicative cycle. The confirmation of these effects would require additional experiments at different concentrations, which were beyond the scope of this work.

**Figure 8:**
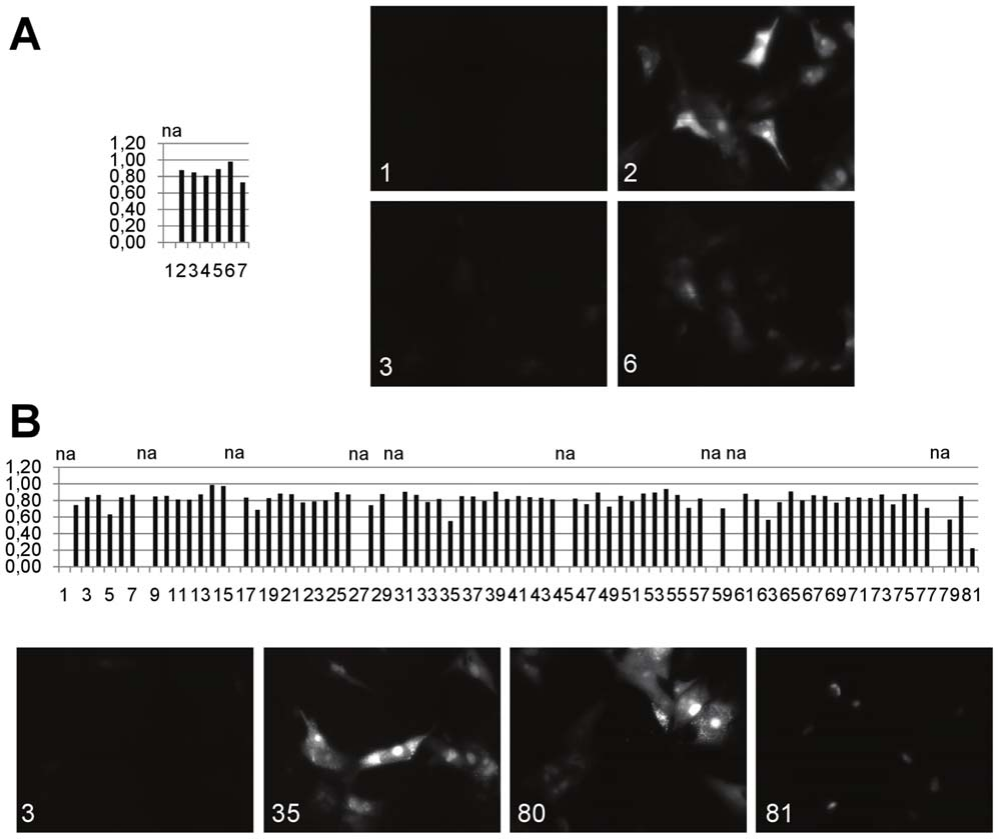
High content analysis of nucleocytoplasmic shuttling of ppUL83-EYFP. Human foreskin fibroblasts were infected with TB4-UL83-EYFP and treated with different concentrations of ganciclovir (A) or kinase inhibitors (B). Fluorescence images were recorded and analyzed in a BD Pathway 855 for nuclear or cytoplasmic localization. (A) In the diagram on the left the ratio of cells with a cytoplasmic/nuclear staining of higher than 0.7 is shown. Infected cell were treated with 100 µM (3), 33 µM (4), 11 µM (5), 3.7 µM (6) or 1.2 µM (7) ganciclovir. Uninfected (1) or infected (2) cells were used as controls. The localization of ppUL83-EYFP in selected samples is shown in the right panel; numbers indicate the respective sample of diagram. (B) The diagram on the top shows the ratio of cells with a cytoplasmic/nuclear staining of higher than 0.7, as above. Kinase inhibitors (1–80) are ordered as in Fig. 7. Leptomycin B (81) was used as positive control. The localization of ppUL83-EYFP in selected samples is shown in the lower panels. Several samples were marked as not applicable (na), since too few cells were above an intensity threshold for EYFP fluorescence.

Finally, we analyzed the nucleocytoplasmic translocation of ppUL83-EYFP on the single cell level (Fig. 8). For this we determined for each sample the percentage of cells showing a cytoplasmic translocation. In samples infected with TB4-UL83-EYFP we observed a translocation to the cytoplasm in more than 80% of the cells (Fig. 8A, sample 2). For mock infected cells (Fig. 8A, sample 1) this type of analysis was not applicable since no EYFP fluorescence was present. Using the nuclear export inhibitor leptomycin B as positive control we detected a reduction to 22% (Fig. 8B, sample 81). For most of the remaining compounds we measured cytoplasmic translocation rates around 70–80%. For three compounds (Tyrphostin 51, GW 5074, SC-514; samples 35, 63, 79) somewhat lower rates of 50–60% were detected. Whether these inhibitors indeed have a direct or indirect role in nucleocytoplasmic translocation of ppUL83 will require more detailed investigations. Several samples were excluded from the analysis since not enough cells could be analyzed. In summary, we could show that all three viruses developed are useful for fast screening of inhibitors of HCMV infection. In addition, the TB4-UL83-EYFP virus can be used to analyze nucleocytoplasmic translocation in a high-content setting.

## Discussion

In this study, we constructed three different recombinant HCMV containing fusions of EYFP to viral proteins of different gene expression kinetic classes. We could show that all recombinant viruses grew similar to wild-type and observed no disturbance of protein expression and intracellular localization. In consideration of the urgent need to find new antiviral agents for the treatment of patients, we established a new method to measure the effect of antivirals in intact cells rather than fixated cells or cell extracts.

To generate the recombinant viruses we developed a vector, where EYFP was placed next to a kanamycin resistance marker, to be able to select for transfer. Both genes were amplified with primers containing sequences homologous to the desired insertion site. The kanamycin gene was flanked by FRT-sites, which were then used by FLP recombinase to remove the resistance marker. As a result, EYFP was integrated into the viral genome and one FRT site along with flanking vector sequences of a total 88 bp remained after the marker excision. Previously we had used a similar strategy for re-insertion of a deleted gene into a deletion mutant of HCMV [34]. This strategy allowed the successful and straightforward fusion of EYFP to the C-terminus of three viral proteins.

So far only few recombinant herpesviruses encoding fluorescent proteins have been described [16], [17], [23], [35]. For HCMV, recombinant viruses expressing EGFP from HCMV enhancer/promoter were generated for different strains and used for screening antivirals [22]–[24], [36]. Fusion proteins have been generated by fusing EGFP with IE2 and ppUL32 (pp150), but only the ppUL32-fusion has been analyzed in detail [26]. In our study we generated fusions of EYFP with three viral proteins, IE2, ppUL83 and ppUL32. All recombinant viruses were viable and grew to the same extent as the wild type virus. For the ppUL83-EYFP fusion this was expected, since the deletion of UL83 did not affect viral growth in fibroblasts. However, both IE2 [6], [7] and UL32 [13], [14], [37], [38] are essential genes. Our observation that both viruses with fusion of EYFP fused to IE2 or UL32 did not show any growth defect or slowdown of the replicative cycle implies that no essential function of these proteins is affected in HFF.

We first analyzed whether the recombinant viruses could be used for investigations of the dynamics of virus infection. For this the fusion proteins needed to be expressed and localized correctly. Since EYFP is a spectral variant derived from EGFP by a few point mutations we could use an anti-EGFP antibody to detect EYFP. By western blot experiments we could detect the fluorescent fusion partner in all recombinant viruses by using either a specific antibody or an antibody directed against GFP. In all cases a shift in protein size of about 26 kDa, the size of EYFP, was observed. Only in the case of ppUL83 a shorter specific band of about the size of native ppUL83 was observed. In principle, such a form could arise by incomplete synthesis or alternatively by post-translational processing. The latter case would generate free EYFP, which would be detrimental to the use of the virus for analysis of intracellular dynamics. However, the anti-EGFP antibody did not detect any free EYFP, favouring the first hypothesis, namely incomplete synthesis. In the next step, we analyzed the localization of the EYFP-fusion proteins. We compared both the intracellular localization in wild type and recombinant virus, and the fluorescence distribution arising from EYFP and the viral protein stained by specific antibodies. In all cases a close overlap was observed. IE2 showed a purely nuclear staining, as reported [4]. ppUL83 was localized to the nucleus during the early phase of infection and moved to the cytoplasm in the late phase [39]. At immediate-early times a punctate pattern on the cell surface could be detected, potentially due to adhered viral particles. ppUL83 from infecting particles accumulated in the nucleus and could be clearly detected by antibody staining. In contrast, EYFP-fluorescence was much weaker, which can be explained by the fact that in particles of TB4-UL83-EYFP only part of ppUL83 is fused to EYFP as shown by immunoblot. Finally, during the late phase we could detect ppUL32 only in the cytoplasm [40]. In summary, the fusion of EYFP to the viral proteins did not affect their respective expression or localization, which makes these recombinant viruses useful tools for the analysis of dynamic processes during viral infection.

As application we explored the use of these recombinant viruses to measure the potency of different antiviral agents against the human cytomegalovirus. We could establish conditions suitable to measure fluorescence intensity non-destructively in live cells and could show that the relative fluorescence intensity is highly correlated to the amount of virus (multiplicity of infection, MOI). Previous experiments using fluorescent HCMV required lysis of cells, which makes these measurements more labour-intensive [22], [41]. To our knowledge, this is the first demonstration of measurement of antiviral effectiveness in intact cells using fluorescent virus. In addition, we were able to reduce the time until measurement from 7–8 dpi as previously described [22], [41] or initially observed (Fig. 6) to 4 dpi.

To test different antiviral substances we first infected HFF and investigated the efficiency of ganciclovir to inhibit viral replication. Ganciclovir (GCV) is a potent antiviral agent which is used for treatment in patients with acute HCMV infection and disease [42]. After treatment with GCV we found an inhibition for all three viruses with an EC50 of 5.4–8.0 µM, which is close to the EC50 of wild type virus, as previously determined [43]. In summary, using our fluorescent HCMV variants we were able to determine correct inhibitory concentrations for GCV and ACV using a non-destructive measurement in intact cells.

To investigate a different mode of antiviral intervention, we also measured the capability of neutralizing antibodies to block the infection using our marked viruses. Since neutralizing antibodies act at the stage of virus attachment or entry we used the recombinant virus TB4-IE2-EYFP. For neutralization we used an approved hyperimmune serum, Flebogamma^R^, as well as supernatants from hybridoma cell lines directed against the viral glycoproteins gH or gB, respectively. We could clearly measure a neutralising capacity of Flebogamma^R^ and the monoclonal antibody 14-4B (anti-gH) [44]. As expected, no neutralizing activity was detected for 27–39 (anti-gB) [45]. These results demonstrate that this fluorescent virus could also be used for screening antiviral agents blocking entry, which have gained attention since the licensing of Enfuvirtide as first viral entry inhibitor for HIV treatment [46].

Finally, we performed a pilot screen using 80 kinase inhibitors. We observed strong inhibition with two compounds (Fig. 7B, samples 2, 3) in cells retaining almost full viability. One compound was Tyrphostin 9, an inhibitor of PDGF receptor kinase [47], which has recently been shown to be necessary for HCMV entry [48]. Tyrphostin 9 had also been described to inhibit Herpes Simplex Virus type 1 replication [49]. The other compound was Rottlerin, which was originally described as PKC inhibitor but might instead be directed towards other kinases [50], [51]. Given these uncertainties, it can only be noted that PKC is required for cytomegalovirus nucleocapsid egress from the cell nucleus by phosphorylation of lamin B [52] and Rottlerin has been shown to block this phosphorylation in the context of Herpes Simplex Virus Type 1 infection [53]. Additional compounds, as KN-93 and Kenpaullone (samples 15, 74), appeared to be more effective in reducing fluorescence from TB4-UL83-EYFP and TB4-UL32-EYFP viruses compared to TB4-IE2-EYFP. Kenpaullone as an inhibitor of Cyclin-dependent kinases 1 and 2 (CDK1 and 2) [54] and glycogen synthase kinase-3b (GSK-3b) [55] could be expected as inhibitor of HCMV replication, since it has been shown that inhibition of CDK2 activity blocks HCMV replication and late gene expression [56]. In our measurements the effect of Kenpaullone on the late expressed ppUL32-EYFP appeared to be most pronounced. This indicates that the viruses could also be used for the discrimination of effects on different phases of viral replication. However, an in depth analysis of the identified compounds would require additional and more detailed experiments. As additional application, we established conditions for high content screening to detect the cytoplasmic translocation of ppUL83-EYFP during the late phase. We could clearly discriminate infected, untreated cells from infected cells treated with Leptomycin B, which was previously reported to block the translocation to the cytoplasm [11]. However, we could not identify additional compounds blocking this nucleocytoplasmic translocation among the screened kinase inhibitors. The fact that we could identify several kinase inhibitors whose targets have been shown to be essential for HCMV replication make it clear that these viruses are valuable tools for antiviral screening.

When we compared the different types of fluorescent viruses, we observed that TB4-IE2-EYFP and TB4-UL83-EYFP produced the highest fluorescence intensity and are best used when high sensitivity is required. Both viruses readily allowed antiviral screening to be performed as early as 4 dpi. In an antiviral screening setting the comparison of the three viruses will give a first indication where in the virus life cycle an inhibiting compound acts. We observed that some substances affected fluorescence intensity of TB4-UL83-EYFP and TB4-UL32-EYFP to a greater extent than that of TB4-IE2-EYFP. Since UL83-EYFP and UL32-EYFP are expressed at a later time point than IE2-EYFP it is plausible that these substances act during the early or late phase. This is an improvement compared to an HCMV strain previously used for antiviral screening [22]. Finally, the fact that UL83-EYFP makes a nucleocytoplasmic translocation at the transition from early to late phase it is possible to use this virus to study the inhibition and dynamics of this process.

In conclusion, we generated and characterised successfully recombinant human cytomegaloviruses with fusions of EYFP to proteins of three different kinetic classes which can be used for antiviral screening. For this task, we established conditions for the non-destructive measurement in intact cell culture models. We believe that these viruses will be highly useful tools to investigate the intracellular dynamics during infection. The viruses will also be valuable tools for time-efficient screening in intact cells and for more advanced screening strategies, such as high content screening [57].

## Materials and Methods

### Oligonucleotides

Oligonucleotides were purchased from Biomers (Ulm, Germany). All oligonucleotides are noted in 5′ to 3′ direction.

IE2-3gfpBAC 5′-TGAGCCTGGCCATCGAGGCAGCCATCCAGGACCTGAGGAACAAGTCTCAGGGCCCCGGCCCCATGAATTCACTGATCAAGGA-3′, IE2-ko5-BAC 5′-CGGGGAATCACTATGTACAAGAGTCCATGTCTCTCTTTCCAGTTTTTCACCGTCGTGGAATGCCTTCGAATTC-3′, ul83-egfp-BAC 5′-ACGCCTTGCCCGGGCCATGCATCGCCTCGACGCCCAAAAAGCACCGAGGTGGCCCCGGCCCCATGAATTCACTGATCAAGGA-3′, UL83-ko5 5′-AGTGGACGTGGGTTTTTATAGAGTCGTCCTAAGCGCGTGCGGCGGGTGGCCGTCGTGGAATGCCTTCGAATTC-3′, ul32-egfpBAC 5′-CCGTGCAGAACATCCTCCAAAAGATCGAGAAGATTAAGAACACGGAGGAAGGCCCCGCGGCCATGAATTCACTGATCAAGGA-3′, ul32-ko5-gfp 5′CACTATCCGATGGTTTCATTAAAAAGTACGTCTGCGTGTGTGTTTCTTAACGTCGTGGAACTTCGAATTC-3′

### Construction of Recombinant Viruses

To generate a suitable recombination vector, we excised the EYFP gene from pEYFP-C1 (Clontech, Heidelberg, Germany) and cloned it into the vector pSL-FRT [58], [59] using restriction sites *Sal*I and *Nhe*I. The resulting plasmid, pSL-FRT-Kan-EYFP, contained the EYFP-gene adjacent to a kanamycin-resistance gene, which was flanked by FRT-sites to allow its removal by FLP-mediated recombination.

To generate linear fragments for recombination, we performed touchdown PCR to amplify the whole EYFP kanamycin-cassette. Primers included 50 nts sequence homologous to the intended insertion site. In addition, a GPGP linker peptide was encoded by the fusion primer between the last codon of the respective viral gene and the first codon of EYFP to ensure conformational uncoupling between the viral protein and the fused EYFP.

The PCR-products were purified (PCR-Purification Kit, Qiagen, Hilden, Germany) and digested with *Dpn*I to remove the template plasmid. The fragments were transformed into *E. coli* strain DH10B (Life Technology, Karlsruhe, Germany) containing the *bacterial artificial chromosome* TB4 (short for TB40E-BAC4) and plasmid pKD46 encoding the γ, β and exo genes of the Red recombinase system [60]. The TB4-BAC was designed to have the BAC cassette inserted into the US2 to US6 gene region, as described in [61]. After selection on kanamycin, the kanamycin resistance gene was removed by FLP-recombinase expressed from plasmid pCP20 [62]. Removal was confirmed by colonie-PCR.

### Cell Culture and Virus Reconstitution

Human foreskin fibroblasts (HFF) were cultivated in minimal essential medium (MEM) (Gibco/BRL, Eggenstein, Germany) supplemented with 10% fetal calf serum, 2 mM L-Glutamine (Biochrom AG, Berlin, Germany), 100 U/ml Penicillin/100 µg/ml Streptomycin (Gibco/BRL). The cells were grown at 37°C with 95% humidity and 5% CO_2_.

For reconstitution, DNA from recombinant BAC clones was purified by alkaline lysis (Midi Prep PC100, Macherey & Nagel, Düren, Germany) and transfected into HFF using the EasyjecT Optima® electroporation system (EquiBio Ltd., Middlesex, UK) with the settings 200 V and 1500 µF. After electroporation cells were seeded into 75 cm^2^ flasks and incubated for 2 weeks to let plaques develop. The successful reconstitution was monitored by fluorescence microscopy. Cell-free virus stocks were produced by ultracentrifugation of the supernatants of infected HFF.

### Southern-Blot Analysis

BAC-DNA was digested with the restriction enzyme *EcoR*V and separated by gel electrophoreses using a 0.6% agarose-gel. For blotting, the gel was washed for 20 min in 0.25 M HCl, rinsed in water followed by incubation for 30 min in 1.5 M NaCl and shortly washed with 0.5 M NaCl. The gel was then equilibrated in transfer buffer (1.5 M NaCl and 0.25 M NaOH) and nucleic acids transferred to a Hybond N nylon membrane (Amersham, Germany) by capillary blot [63]. After over night transfer the DNA was UV-crosslinked to the membrane (CL-1000 Ultraviolet Crosslinker UVP, Cambridge, UK).

Probes for hybridization were generated by PCR amplification and subsequent agarose gel purification. Radioactive labelling was performed by random hexamer priming using Ready-to-go DNA labelling beads (Amersham Pharmacia Biotech, USA) and α^32^P-dCTP. Radioactive hybridizations were performed in a hybridization incubator (Robbins Scientific, Sunnyvale, CA) essentially as described previously [64]. For prehybridization, filters were incubated for at least 4 h at 42°C in a solution containing 50% formamide, 5x SSC (1x SSC is 0.15 M NaCl plus 0,015 M sodium citrate), 50 mM sodium phosphate pH 6.5, 5x Denhardt's solution and 1 mg/ml yeast RNA (Fluka, Taufkirchen, Germany). Hybridizations were performed for at least 24 h in hybridization solution containing 50% formamide, 5x SSC, 20 mM sodium phosphate pH 6.5, 5x Denhardt's solution and 500 µg/ml yeast RNA. Filters were then washed several times with buffer containing 20 mM sodium phosphate, 0.1% sodium dodecyl sulfate (SDS) and decreasing concentrations of SSC. For reprobing of the filters, the radioactive probe was removed by two incubations in 0.1% SDS at 95°C for 30 min. Bound probes were detected on Kodak X-Omat films.

### Virus Growth Kinetics

For virus growth kinetics, 4×10^4^ HFF were seeded into a 24-well plate and infected with a *multiplicity of infection* (MOI) of 1, 0.1 or 0.01 for TB4-wt and all three different variants. After infection the plates were centrifuged for 30 min with 2000 rpm at room temperature and incubated for 3 h after the centrifugation. Thereafter, the cells were washed with PBS and the medium was changed. At day 1, 3, 5, 7 and 10 after infection supernatants were harvested and stored frozen in sucrose phosphate-buffer at −70°C. After the titration using HFF in 96-well plates the *plaque forming units* (pfu) per ml were determined. All titrations were done in triplicates in three independent experiments.

### Immunoblot

HFF were infected with MOI of 1 for 72 hours in 6-well plates and then harvested by scraping the cells off in 500 µl PBS. Cell pellets were resuspended in 5xSDS-Buffer (200 mM Tris, 5 mM EDTA, 1 M sucrose, 0.1% bromphenole blue, 1 mM DTT and pH 8,8) and boiled at 95°C for 5 min. The cell-lysates were separated by using a 10% SDS-PAGE and blotted on a PVDF-Membrane. For detection rabbit serum directed against IE1+2 [65] and GFP (Gentaur, Brüssel, Belgium) or mouse monoclonal antibodies directed against ppUL83 (28–77) [66] and ppUL32 (XP-1), [67] were used. We used goat anti-rabbit or goat anti-mouse HRP-labelled secondary antibodies (Pierce, Ireland).

For the detection of major tegument proteins and fusion proteins in virus particles, supernatant from infected cells was clarified from cellular debris by low speed centrifugation (3000 rpm, 10 min) followed by ultracentrifugation (28000 rpm, 70 min) to concentrate virus particles. Virus stocks were adjusted for equal levels of major capsid protein by titration and immunoblot detection. For detection, rabbit serum directed against GFP (Gentaur, Brüssel, Belgium), ppUL32 (XP1-5) [67] and ppUL82 (M. Winkler, unpublished data) or mouse monoclonal antibodies directed against pUL86 (28-4) [68] and ppUL83 (28–77) [66] were used. We used goat anti-rabbit or goat anti-mouse HRP-labelled secondary antibodies (Pierce, Ireland).

### Immunofluorescence

For immunofluorescence analysis, 4×10^4^ HFF were seeded on glass cover slips in 24-well plates and incubated overnight. Cells were infected with MOI of 0.5 and incubated for 24, 48 and 72 hours. For fixation cells were incubated in 4% PFA for 10 min and 0.1% Triton X-100 for 2 min followed by washing steps in PBS. Viral proteins were detected using specific primary monoclonal mouse antibodies against ppUL83 (Cinapool, Argene), ppUL32 (XP-1) or IE1–2 (E13, Argene). As secondary antibody we used Alexa555-labelled polyclonal goat anti-mouse antibody (Invitrogen).

### Antiviral Screening

To assess the usefulness of the TB4-IE2-EYFP, TB4-UL83-EYFP or TB4-UL32-EYFP virus variants for detection of antiviral activity, we performed infection experiments in 96-well plates. We seeded 1.7×10^4^ HFF per well in culture media (as described before) and incubated at 37°C over night. To measure the relation between multiplicity of infection and fluorescence intensity, we measured different MOI after 8 days of incubation. For this experiment the cells were infected with MOI of 10, 3, 1, 0.3, 0.1 and 0.01.

To test viral sensitivity for antiviral agents, cells were infected with TB4-IE2-EYFP, TB4-UL83-EYFP or TB4-UL32-EYFP at MOI of 0.1. Ganciclovir (GCV, Cymeven, Syntex, Germany) was added to the infected cells using different concentrations. After 9 days of incubation at 37°C, cells were washed and incubated in PBS for the measurement. PBS gave the best results compared to medium without pH-indicator or normal medium. Relative fluorescence intensity was measured using a fluorescence spectrophotometer (Cary Eclipse, Varian, Darmstadt, Germany). Using excitation and emission slit width of 10 nm the emission was scanned from 500 nm to 600 nm at an extinction setting of 480 nm.

To measure neutralization by antibodies, TB4-IE-EYFP (MOI of 0.1) was pre-incubated with Flebogamma (5%; Grifols, Germany), mouse monoclonal anti-gH (14-4B) [44] or mouse monoclonal anti-gB (27–39) [45] for 1 hour at 37°C and 15 min at 4°C. After incubation we transferred the pre-incubated virus-antibody mix to the cells and measured the relative fluorescence intensity again after 8 days.

For screening a compound library, we seeded 1.7×10^4^ HFF per well in 96-well plates in culture media and incubated at 37°C over night. Cells were infected with fresh supernatant of TB4-IE2-EYFP, TB4-UL83-EYFP or TB4-UL32-EYFP. Virus supernatant was titrated in parallel and corresponded to a MOI of 0.3 to 1. As compounds we used a kinase inhibitor library (Enzo, Loerrach, Germany); each inhibitor was tested in triplicates. Inhibitors were added together with virus at a final concentration of 10 µM and were replaced every second day. For measurements cell culture medium was removed and cells were washed twice in PBS. Measurements were taken in a Safire 2 (Tecan, Crailsheim, Germany) from the top at excitation wavelength 485 nm (20 nm bandwidth) and emission wavelength 525 nm (20 nm bandwidth) with an integration time of 40 µs and 10 reads per well. We used optimal gain, optimized Z-position and high sensitivity flash mode. Background was substracted from raw data and infected cells were normalized to 100%. Calculations and descriptive statistics were carried out in Excel. EC50 values were determined with the IC50 package in the R environment [69]. We use the term EC50 as the FDA recommended this term in 2007 for use in cell-based drug susceptibility testing (http://www.fda.gov/downloads/Drugs/GuidanceComplianceRegulatoryInformation/Guidances/ucm071173.pdf). Cell viability was measured in parallel plates using the colorimetric WST-1 cell proliferation assay (Roche, Mannheim, Germany).

The nucleocytoplasmic translocation of ppUL83-EYFP was analyzed in a Pathway 855 (Beckton-Dickinson; Heidelberg, Germany). Two hours before imaging live cells Hoechst 33342 (Invitrogen, Karlsruhe, Germany) was added at a final concentration of 1 µg/ml to allow delineation of the nuclei. A matrix of 3×3 images was taken for each well (Olympus 20X objective NA 0.75, EYFP: excitation filter 500/20, emission filter 535/30). To define regions of interest, the Hoechst stained nuclei where segmented. To define cytoplasmic localization, a second ring-shaped mask around a nucleus was defined starting 10 pixels away from the nucleus and reaching 10 pixels into the cytoplasm. The gap between the nuclear mask and the ring mask reduces nuclear fluorescence in the cytoplasmic region. The algorithm automatically prevents adjacent ring masks from overlapping. All image processing was performed using the BD-Attovision Software Version 1.6.1. To exclude background staining, we set a threshold for EYFP nuclear staining at an intensity of 450. Samples with less than 40 cells above this threshold were excluded from further analysis (Fig. 8: na, not applicable). Since ppUL83 first accumulates in the nucleus and moves to the cytoplasm in the late phase, we used a ratio of cytoplasmic EYFP/nuclear EYFP of more than 0.7 to count cells as positive for cytoplasmic translocation. The percentage of cells positive for cytoplasmic translocation related to the total number of cells above the intensity threshold was used to measure the rate of translocation.
